# Enhanced Poly(Propylene Carbonate) with Thermoplastic Networks: A One-Pot Synthesis from Carbon Dioxide, Propylene Oxide, and a Carboxylic Dianhydride

**DOI:** 10.3390/polym10050552

**Published:** 2018-05-21

**Authors:** Xianggen Chen, Lingyun Wang, Jiuying Feng, Xianling Huang, Xiuzhi Guo, Jing Chen, Zhenyuan Xiao, Xiangjun Liang, Lijun Gao

**Affiliations:** 1 School of Chemistry and Chemical Engineering, Resource and Chemical Engineering Technology Research Center of Western Guangdong Province, Lingnan Normal University, Zhanjiang 524048, China; cxgzgah@163.com (X.C.); fengjy@lingnan.edu.cn (J.F.); hangxl95@163.com (X.H.); guoxzls@163.com (X.G.); chenjing1@lingnan.edu.cn (J.C.); xiaozhenyuan1996@163.com (Z.X.); xjliang1997@163.com (X.L.); 2 Key Laboratory of Functional Molecular Engineering of Guangdong Province, School of Chemistry and Chemical Engineering, South China University of Technology, Guangzhou 510641, China; lingyun@scut.edu.cn

**Keywords:** poly(propylene carbonate), networks, dianhydride, terpolymerization, modification

## Abstract

Thermally and mechanically enhanced poly(propylene carbonate) (PPC) with networks was prepared by adding a cyclic carboxylic dianhydride, bicyclo(2,2,2)oct-7-ene-2,3,5,6-tetracarboxylic dianhydride (BTCDA), in the CO_2_/propylene oxide (PO) copolymerization. The obtained copolymers were characterized by FT-IR, ^1^H NMR, DSC, and TGA. The gel, melt flow rate, hot-set elongation, and tensile properties were also measured. The formation of networks was confirmed by the presence of gel and the shape recovery after the hot-set elongation test. The minimum permanent deformation of the copolymer is 3.8% and that of PPC is 4539% higher than this value. The results show that BTCDA units are inserted into the backbone of PPC, and the PPC chains are connected successfully owing to cyclic multifunctional anhydride groups in BTCDA. With increasing feed molar ratio of BTCDA to PO from 1 to 4%, the yield strength of copolymers increases from 18.1 to 37.4 MPa compared to 12.9 MPa of PPC. The 5% weight-loss degradation temperatures and maximum weight-loss degradation temperatures greatly increase up to 276.4 and 294.7 °C, respectively, which are 58.6 °C and 55.1 °C higher than those of PPC. These enhanced properties originate from the formation of crosslinks by the rigid and bulky multifunctional dianhydride.

## 1. Introduction

Poly(propylene carbonate) (PPC) from the CO_2_/propylene oxide (PO) catalyzed copolymerization, as a biodegradable polymer, has been drawing much attention in both academic and industrial fields. It has a wide range of potential applications, such as binders, brazing pastes and solutions, propellants, and diamond cutting tools [1]. In the past decades, great efforts were devoted to develop and commercialize the catalysts [2,3,4,5,6,7,8,9,10]. However, PPC still has considerable shortcomings, such as its low decomposition temperature and low glass transition temperature (*T*_g_), that severely limit its thermal stability and practical application as a viable biodegradable plastic [11,12]. Thus, the reinforcement of PPC is in urgent demand to extend its applications. Crystallization may enhance PPC, but it is difficult to crystallize PPC even though stereogradient PPC has been synthesized [10,13], and stretching does not make PPC crystallize, although aliphatic polycarbonates of similar structure to PPC, such as poly(cyclohexene carbonate) or poly(trimethylene carbonate), can crystallize by stereoregularization or stretching [14,15]. Up to now, many attempts, such as terpolymerization with co-monomers [16,17,18,19], crosslinking [20,21,22], and fabrication with other polymers [23,24,25,26,27], inorganic fillers [28,29,30,31,32], or organic compounds [33,34,35,36] have been carried out to improve the thermal and mechanical properties. In addition, PPC-inorganic hybrid materials like PPC grafted multi-walled carbon nanotubes were prepared [37]. In fact, several efficient strategies have been achieved. Nevertheless, the difficulty in the modification of PPC is how to comprehensively improve its properties under the premise that the second component is introduced in a very small amount, otherwise the significance of using CO_2_ resource is weakened. It is worth noting that both physical and chemical crosslinking has become a quite effective modification method for PPC. For example, a small quantity (1 wt %) of graphene oxide (GO) nanosheets can greatly reinforce PPC. The uniform dispersion and physical crosslinking of GO in PPC matrix were considered as the main mechanism [28]. Adding 2.5 wt % of hyperbranched poly(ester-amide) (HBP) into PPC also obviously enhanced PPC, in which the formed hydrogen bonds between many hydroxy/amino groups in HBP and carbonyls in PPC played a key role [12]. Here, the physical crosslinking of hydrogen bonds is clear. What’s more, the crosslinked PPC exhibited very good thermal stability and mechanical strength, especially excellent dimensional stability at elevated temperature despite having a low *T*_g_ [20], that can effectively solve the issue of cold flow for PPC. Crosslinking customarily requires two steps including introducing a crosslinkable moiety like double bonds into the PPC backbone and subsequently crosslinking using radical initiators. It was also reported that crosslinked PPC can be obtained by the terpolymerization of CO_2_, PO, and diepoxide [22,38]. However, the addition of diepoxide in the CO_2_/PO copolymerization did not always form crosslinked PPC [39,40].

Cyclic anhydrides can terpolymerize with CO_2_ and PO [41,42,43,44,45,46]. But, reports of directly generating crosslinked PPC through adding dianhydride in the CO_2_/PO copolymerization are rare. In previous work [47], we prepared the pseudo-interpenetrating poly (propylene carbonate) networks by terpolymerization of CO_2_, PO, and pyromellitic dianhydride (PMDA). In relevant work, Song and coworkers prepared crosslinked PPC by CO_2_/PO/itaconic anhydride terpolymerization [48]. Hilf and coworkers prepared it using two-step method in which PPC with furyl pandants was first synthesized and then crosslinked by dianhydride crosslinkers [49], but there were no mechanical properties given. We recently also prepared the similar crosslinked PPC through Diels–Alder reaction between PPC chains separately containing maleic anhydride units and furyl pandants. It is amazing that the mechanical strength not only did not rise, but reduced although PPC was crosslinked. This phenomenon has been not observed about crosslinked PPC up to now. So, we infer that the bulk and rigid properties of the crosslinker play an important role in improving PPC’s performance. Here, we try to introduce a cyclic dianhydride with bridged-ring structure, bicyclo(2,2,2)oct-7-ene-2,3,5,6-tetracarboxylic dianhydride (BTCDA), into the PPC chains which are supposed to be connected by the former. The thermal and mechanical properties and dimensional stability were fully investigated in this work.

## 2. Materials and Methods

### 2.1. Materials

PO was refluxed over calcium hydride for 8 h, distilled under dried nitrogen gas, and stored over 0.4 nm molecular sieves prior to use. CO_2_ of 99.99% was commercially obtained without further purification. BTCDA was purchased from Aladdin Industrial Corporation and used without further purification. Zinc glutarate (ZnGA) was synthesized according to Ref. [2]. All other reagents and solvents were of analytical grade and used without further purification.

### 2.2. General Copolymerization Procedure

The terpolymerization were carried out in a 100 mL autoclave reactor equipped with a magnetic stirrer. The pre-dried 0.1 g of ZnGA catalyst and a proportion of BTCDA were put into the autoclave and were dried under vacuum at 100 °C for 8 h. Then the autoclave was cooled to under 15 °C and was purged carefully with N_2_ and evacuated alternatively three times, followed by injecting 30 mL PO with a syringe. The autoclave reactor was then pressurized to 5.0 MPa via a CO_2_ cylinder. The terpolymerization was performed at 60 °C under stirring for 40 h. Afterwards the autoclave was cooled to room temperature and the pressure was released. The hard-lump product was dissolved in a sufficient volume of chloroform containing 5% solution of hydrochloric acid to decompose the catalyst. The organic layer was washed into the neutral reaction and slowly added to excess, while vigorously stirred with ethanol to precipitate the copolymers. Dissolution and precipitation of the copolymers was carried out repeatedly to remove a small quantity of cyclic propylene carbonate and unreacted BTCDA until they gave no ^1^H NMR signals. Then, the copolymers were dried at 80 °C under vacuum to a constant weight and the yields were calculated.

PPC was also synthesized in a similar procedure to that of the copolymers, except that no BTCDA was added to the autoclave. 

### 2.3. Characterization and Measurements

Fourier transform infrared spectroscopy (FT-IR) measurements were performed on a Thermo Scientific Nicolet 6700 spectrometer (Madison, WI, USA) equipped with an attenuated total reflection (ATR) accessory.

^1^H NMR spectra were measured using a Bruker DRX-400 spectrometer (Rheinstetten, Germany) with deuterated chloroform as solvent.

The gel contents were measured according to ASTM D2765 using a Soxhlet extractor. A sample was refluxed in boiling chloroform for 24 h. The insoluble portion was dried to a constant weight at 80 °C under vacuum. The gel content was defined as the percentage of the weight of insoluble portion in the sample. The data were recorded as the average value of three parallel determinations.

The thermogravimetric analysis (TGA) measurements were performed on a PerkinElmer simultaneous thermal analyzer (Model STA 6000, Waltham, MA, USA). The samples were tested under nitrogen flow of 40 mL·min^−1^ from 25 to 400 °C at a heating rate of 20 °C·min^−1^.

The differential scanning calorimetry (DSC) measurements were conducted using a Q100 TA instrument (New Castle, DE, USA) under a high purity nitrogen flow over the temperature range from −25 to 200 °C at a heating rate of 10 °C·min^−1^. The *T*_g_ of the sample was taken as the onset of the change in heat capacity as a function of temperature. 

The tensile tests were conducted using a CMT 6104 testing machine (MTS industrial systems (China) Co., Shenzhen, China) according to ASTM D638. The cross-head speed was 50 mm·min^−1^. The data were recorded as the average value of five parallel determinations. The dumbbell-shaped specimens were prepared by hot-pressing into sheets, followed by cutting using a dumbbell cutter. Prior to measurements, the specimens were conditioned at 23 °C and 50% ± 5% humidity for 24 h.

The melt flow rate (MFR) measurements were carried out using a melt index meter (JQ-400A, Jian Qiao Test Equipment Co., Dongguan, China) according to GB/T 3682-2000. The temperature and the load of the test process were 170 °C and 2.16 kg, respectively. The data were recorded as the average value of four parallel determinations.

The hot-set elongation tests were carried out in an oven. The dumbbell-shaped specimen was loaded under 0.14 MPa and marked as reference length *L*_0_ (*L*_0_ = 20 mm). The loaded specimen was placed in an oven at 60 °C, and the length *L*_1_ between the two markers was measured after 15 min. Then the load was released and the specimen was allowed to relax at 60 °C for 5 min. Afterwards the specimen was taken out of the oven and continued to be cooled to room temperature. The length *L*_2_ was measured. The hot-set elongation and permanent deformation were calculated as (*L*_1_ − *L*_0_)/*L*_0_ × 100% and (*L*_2_ − *L*_0_)/*L*_0_ × 100%, respectively.

## 3. Results and Discussions

### 3.1. Synthesis

**Scheme 1:**
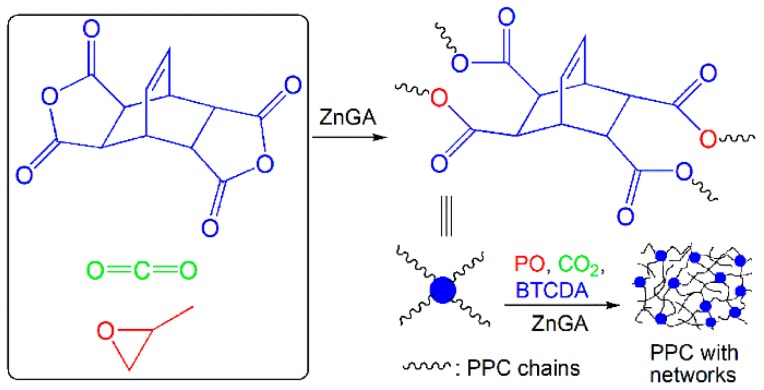
The one-pot synthesis of poly(propylene carbonate) (PPC) with networks by CO_2_/PO/BTCDA terpolymerization.

As expected, owing to the multifunctional anhydride groups of BTCDA, a unit of BTCDA linked to four PO/CO_2_ copolymer arms forms once BTCDA enters into the reaction upon introducing BTCDA in the PO/CO_2_ copolymerization. Random joining of these many units produces connected PPC chains (Scheme 1). The results of yield and gel content measurements indicate that the addition of BTCDA has a considerable influence on increased gel content and an adverse one on decreased yields of copolymers (Table 1). When BTCDA increased from 0 to 4 mol % of PO, the yields of copolymers decrease modestly, while the gel content of copolymers increases from 0% to 38.2%. The presence of gel indicates that BTCDA is inserted into the backbone of PPC and the PPC chains are connected successfully.

The FT-IR and ^1^H NMR measurements were used to determine the structure of the copolymers. As shown in Figures S1 and S2, the copolymer has the new characteristic FT-IR absorption peaks at 751 and 698 cm^−1^ that are attributed to the out-of-plane bending vibration of C–H from the *cis*-disubstituted alkene derived from BTCDA units [50]. The peaks at 2990, 1739, 1455, 1400, 1229, 1165, 1070, 976, and 787 cm^−1^ are similar to those of PPC and they are ascribed to the carbonyl and open ring of propylene oxide [47]. It indicates that the units of BTCDA were incorporated into PPC successfully. The copolymers have the same ^1^H NMR peaks as PPC (δ, ppm): 4.94 (s, CH), 4.13~4.19 (m, CH_2_), and 1.30 (s, CH_3_) [2]. They are attributed carbonate linkages formed by alternating CO_2_/PO copolymerization. In addition, there are two peaks at 3.4~3.6 and 1.1 ppm (Figure S3), which are signals of CH, CH_2_, and CH_3_ from ether linkages, respectively [2]. Based on the integration of methyl groups from the carbonate linkages (δ1.3 ppm) and from ether linkages (δ1.1 ppm), respectively, the contents of ether linkages for PPC and PPC-1~PPC-4 are 5.7, 3.3, 3.0, 2.7, and 2.7 percent. This implies that the incorporation of BTCDA into PPC backbones can repress the continuous insertion of PO monomers into growing chains, resulting in the reduction in ether linkages. However, the absence of ^1^H NMR signals from BTCDA units shows that BTCDA units are mostly limited in the gel which is insoluble in CDCl_3_ solvent. In addition, there were no ^1^H NMR peaks at 6.38 ppm from the hydrogen of carbon=carbon double bond in the BTCDA unit [50], and a methyl appeared at 1.50 ppm in cyclic propylene carbonate [50]. This shows that the unreacted BTCDA and byproduct cyclic propylene carbonate were removed in the purification.

### 3.2. Thermal Properties

**Figure 1:**
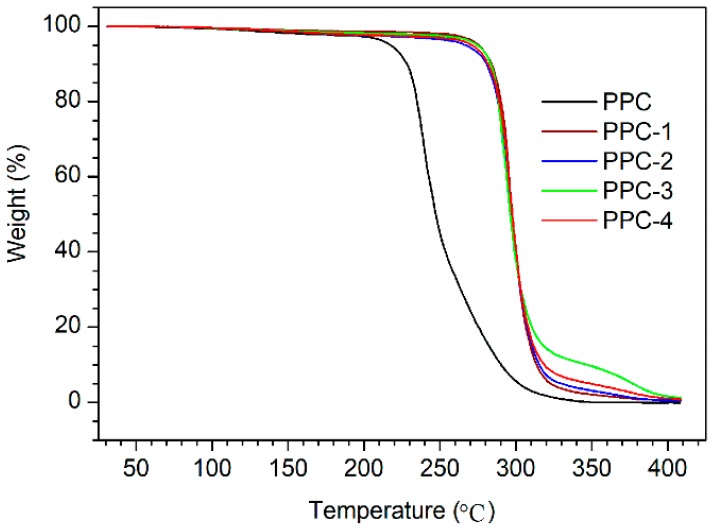
The thermogravimetric analysis (TGA) curves for PPC and PPC with networks.

**Figure 2:**
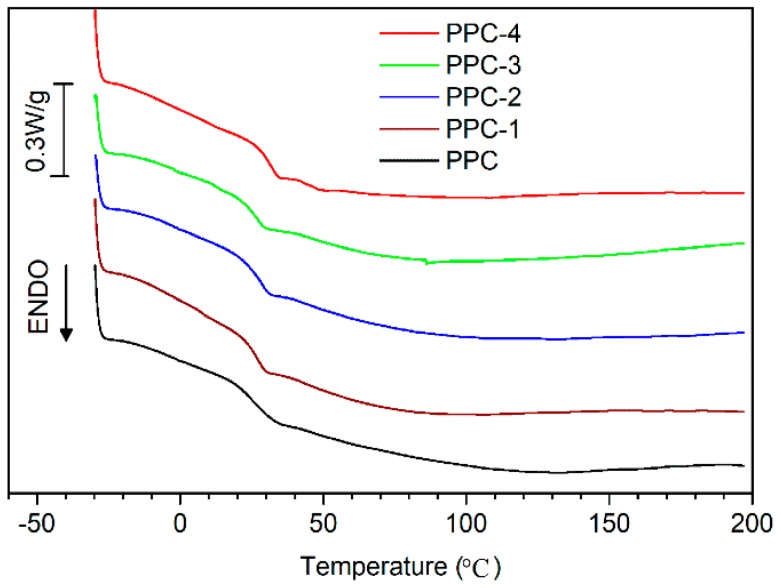
The differential scanning calorimetry (DSC) curves for PPC and PPC with networks.

TGA measurements show that the copolymers can enhance the thermal stability significantly. As shown in Figure 1 and Figure S4, for PPC, the 5% weight-loss degradation temperature (*T*_d,__−5%_) is 217.8 °C, and there are two maximum weight-loss degradation temperatures (*T*_d,max_s) of 239.6 and 255.4 °C. According to the decomposition mechanism of PPC investigated using thermogravimetric analysis/infrared spectrometry techniques [51], the above two *T*_d,max_s arise from chain scission and unzipping reactions, respectively. The *T*_d,__−5%_ and *T*_d,max_ of each copolymer is over 270 and 290 °C, respectively. This great improvement in thermostability is attributed to the formation of crosslinks in the PPC matrix through introducing BTCDA into PPC chains, even when the lowest BTCDA feed concentration (1 mol % of PO) was used, because the crosslinks obviously restrict the unzipping reaction. It is seen that both the *T*_d,__−5%_ and *T*_d,max_ change little with increasing BTCDA feed (Table 2). It indicates that the decomposition temperatures of copolymers don’t increase indefinitely with increasing BTCDA content. That is, when BTCDA feed contents increase from 0 to 1 mol % of PO, the decomposition temperatures should increase gradually at first or from a certain content and become constant thereafter. It is enough to use a small amount of BTCDA, at least below 1 mol% of PO, to focus on the improvement of thermal stability, although it is significant to study the thermal stability at lower BTCDA feed contents and find the maximum BTCDA feed content when the decomposition temperatures begin to gently increase. But, the combined effect on the tensile properties is that BTCDA content below 1 mol% is not enough to enhance the tensile strength, so the terpolymerization using lower BTCDA feed content was not conducted. On the other hand, the glass transition temperatures (*T*_g_s) of PPC and copolymers are in the range of about 25~30 °C and have little difference (Figure 2 and Table 2). It is noteworthy that PPC-4 has another *T*_g_ of 45.2 besides 30.3 °C. Obviously, the low *T*_g_ arises from the CO_2_/PO copolymer chains and the high one arises from the chains containing a high content of BTCDA units.

### 3.3. Mechanical Properties

The mechanical properties of the copolymers are shown in Table 3 and Figure S5. The tensile strength of copolymers can be significantly improved by introducing BTCDA. With increasing BTCDA from 1 to 4 mol %, the tensile strength increases from 18.1 to 37.4 MPa, compared to 12.9 MPa of PPC. Accordingly, the increase of BTCDA content reduces the ductility and toughness of the copolymers. Overall, PPC-2 possesses comprehensive performance with a tensile strength of ~30 MPa and an elongation at break of ~98 percent.

### 3.4. Hot-Set Elongation and Permanent Deformation

It is known that PPC shows very poor heat resistance above *T*_g_ and it will soften while being held in hand. Nevertheless, maintaining the dimensional stability of PPC at above 60–70 °C is crucial for many applications. As shown in Table 4, the hot-set elongation and permanent deformation of PPC is 337.5% and 176.3%, respectively. With increasing BTCDA, the hot-set elongations reduce sharply to 19%, indicating that the incorporation of BTCDA has a considerable influence on heat resistance. What’s more, the permanent deformations greatly reduce from 176.3% to 3.8% (Figure S6). These phenomena also prove that the networks form in the copolymers and these networks enable the copolymers to have stronger resistance to strain and deformation at higher temperatures than PPC.

### 3.5. Rheological Properties

**Figure 3:**
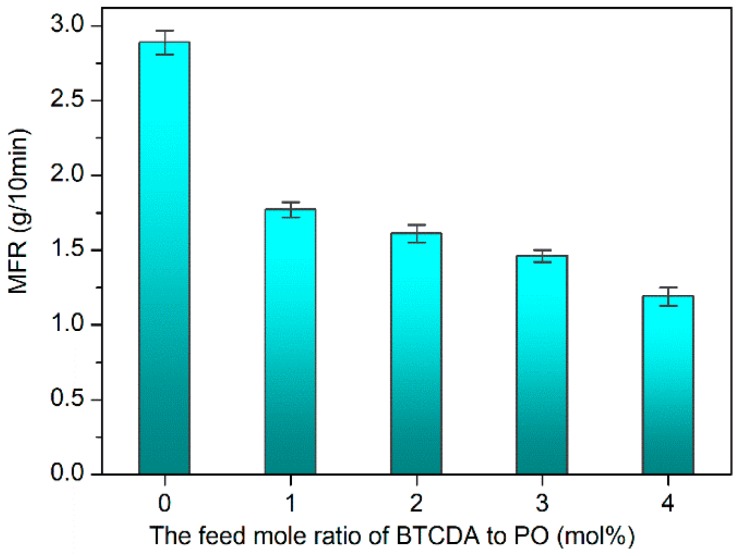
The melt flow rate (MFR) of PPC and PPC with networks.

The rheological performance of polymers is important for optimizing the processing conditions. Therefore, MFR measurements were conducted to study the rheological properties of copolymers. As shown in Figure 3, the addition of BTCDA has an apparent influence on the MFRs of copolymers. The MFR of PPC is 2.89 g/10 min, and those of the copolymers reduced from 1.77 to 1.19 g/10 min with increasing BTCDA. First, we tried to make dumbbell-shaped copolymer specimens for the tensile tests by using an injection machine, but it was not easy to deal with the depression on the surface of the specimens under injection conditions, whereas PPC is allowed for injection molding easily. However, the crosslinking degree enabled the copolymers to be hot-pressure molded even though the PPC chains are connected by BTCDA. The crosslinked PPC modified by 4,4′-diphenylmethane diisocyanate can also be thermoplastic and be hot-pressed by controlling the degree of crosslinking [52].

## 4. Conclusions

Focusing on improving the thermal and mechanical properties and dimensional stability of PPC, we present a one-pot synthesis of PPC with networks by introducing a cyclic multifunctional carboxylic dihydride, BTCDA, in the PO/CO_2_ copolymerization. The networks are confirmed by the presence of gel and the great reduction of permanent deformation. The thermal and mechanical properties and dimensional stability of copolymers were extensively improved owing to the networks generated by introduction of BTCDA, and they are summarized in Table 5 and compared with those reported by other groups. In addition, this PPC with networks is thermoplastic and can be hot-processed by controlling the degree of crosslinking using various BTCDA feed contents.

## Figures and Tables

**Table 1:** The results of yields and gel contents of PPC and PPC with networks.

Sample	Feed Molar Ratio of BTCDA to PO (%)	Yield (g Copolymer/g ZnGA)	Gel (%)
PPC	0	28.2	0
PPC-1	1	25.4	11.4 ± 1.1
PPC-2	2	23.0	14.6 ± 1.3
PPC-3	3	22.5	30.3 ± 2.0
PPC-4	4	22.8	38.2 ± 2.3

**Table 2:** The thermal properties of PPC and PPC with networks.

Sample	*T*_d,−5%_ (°C)	*T*_d,max_ (°C)	*T*_g_ (°C)
PPC	217.8	239.6, 255.4	25.7
PPC-1	271.9	294.4	26.3
PPC-2	274.4	293.5	26.3
PPC-3	275.3	294.9	26.0
PPC-4	276.4	294.7	30.3, 45.2

**Table 3:** The mechanical properties of PPC and PPC with networks.

Sample	Tensile Strength/MPa	Young’s Modulus/MPa	Elongation at Break/%
PPC	12.9 ± 1.6	1233 ± 17	652.3 ± 16
PPC-1	18.1 ± 1.3	1465.4 ± 21	413.8 ± 13
PPC-2	29.5 ± 1.8	1932.2 ± 13	97.8 ± 5
PPC-3	31.9 ± 1.5	2329.2 ± 18	60.9 ± 3
PPC-4	37.4 ± 1.6	2491.5 ± 23	7.4 ± 1

**Table 4:** The results of the hot-set elongation tests.

Sample	Hot-Set Elongation (%)	Permanent Deformation (%)
PPC	337.5	176.3
PPC-1	71.5	38.8
PPC-2	55.0	16.3
PPC-3	26.5	6.3
PPC-4	19.0	3.8

**Table 5:** Properties comparison of crosslinked PPCs prepared in this paper and reported by other groups.

Crosslinking Method ^a^	Feed Content ^b^	*T*_d,−5%_ (°C)	*T*_d,max_ (°C)	Tensile Strength (MPa)	Elongation at Break (%)	Hot-Set Elongation (%)	Permanent Deformation (%)	MFR (g Copolymer/10 min)
CO_2_/PO/AGE terpolymerization followed crosslinked by UV-radiation [20]	5:100	220.5	233.9	- ^d^	-	17.3	0	-
CO_2_/PO/MA terpolymerization followed crosslinked by DCP [21]	1:30	261	300	45.6	-	-	-	-
CO_2_/PO/MDI terpolymerization [52]	1.5:100 ^c^	242	275	36.9	12	-	-	-
CO_2_/PO/PMDA terpolymerization [47]	3:100	281	306	41	406	-	-	-
CO_2_/PO/IAn terpolymerization [48]	5:100	251.3	284.7	27.5	554.6	-	-	-
CO_2_/PO/BTCDA terpolymerization	2:100	274.4	293.5	29.5	97.8	55	16.3	1.61
	4:100	276.4	294.7	37.4	7.4	19	3.8	1.19

^a^ MDI: 4,4′-diphenylmethane diisocyanate; MA: maleic anhydride; DCP: dicumyl peroxide; AGE: allyl glycidyl ether; Ian: itaconic anhydride. ^b^ The feed molar ratio of third monomer to PO except where noted. ^c^ The feed weight ratio of MDI to PO. ^d^ No data reported.

## Supplementary Materials

The following are available online at http://www.mdpi.com/2073-4360/10/5/552/s1, Figure S1: The FT-IR spectrum of PPC, Figure S2: The FT-IR spectrum of PPC-3; Figure S3: The ^1^H NMR spectra of PPC and PPC with networks. Figure S4: The DTG curves for PPC and PPC with networks. Figure S5: The strain-stress curves for PPC and PPC with networks. Figure S6: The photos of dumbbell-shaped specimens before (left) and after (right) hot-set test. (a) PPC, (b) PPC-4. The right photo is the permanent deformation result.

## References

[1] Zhang Z.H., Lee J.H., Lee S.H., Heo S.B., Pittman C.U. (2008). Morphology, thermal stability and rheology of poly(propylene carbonate)/organoclay nanocomposites with different pillaring agents. Polymer.

[2] Ree M., Bae J.Y., Jung J.H., Shin T.J. (1999). A new copolymerization process leading to poly(propylene carbonate) with a highly enhanced yield from carbon dioxide and propylene oxide. J. Polym. Sci. Part A Polym. Chem..

[3] Sheng X.F., Wu W., Qin Y.S., Wang X.H., Wang F.S. (2015). Efficient synthesis and stabilization of poly(propylene carbonate) from delicately designed bifunctional aluminum porphyrin complexes. Polym. Chem..

[4] Klaus S., Lehenmeier M.W., Anderson C.E., Rieger B. (2011). Recent advances in CO_2_/epoxide copolymerization-New strategies and cooperative mechanisms. Coord. Chem. Rev..

[5] Coates G.W., Moore D.R. (2004). Discrete metal-based catalysts for the copolymerization of CO_2_ and epoxides: Discovery, reactivity, optimization, and mechanism. Angew. Chem. Int. Ed..

[6] Poland S.J., Darensbourg D.J. (2017). A quest for polycarbonates provided via sustainable epoxide/CO_2_ copolymerization processes. Green Chem..

[7] Lu X.B., Ren W.M., Wu G.P. (2012). CO_2_ copolymers from epoxides: Catalyst activity, product selectivity, and stereochemistry control. Acc. Chem. Res..

[8] Sujith S., Min J.K., Seong J.E., Na S.J., Lee B.Y. (2008). A highly active and recyclable catalytic system for CO_2_/propylene oxide copolymerization. Angew. Chem. Int. Ed..

[9] Vagin S.I., Reichardt R., Klaus S., Rieger B. (2010). Conformationally flexible dimeric salphen complexes for bifunctional catalysis. J. Am. Chem. Soc..

[10] Nakano K., Hashimoto S.i., Nakamura M., Kamada T., Nozaki K. (2011). Stereocomplex of poly(propylene carbonate): Synthesis of stereogradient poly(propylene carbonate) by regio-and enantioselective copolymerization of propylene oxide with carbon dioxide. Angew. Chem. Int. Ed..

[11] Luinstra G.A., Borchardt E. (2011). Material properties of poly(propylene carbonates). Adv. Polym. Sci..

[12] Chen L.J., Qin Y.S., Wang X.H., Li Y.S., Zhao X.J., Wang F.S. (2011). Toughening of poly(propylene carbonate) by hyperbranched poly(ester-amide) via hydrogen bonding interaction. Polym. Int..

[13] Li B., Wu G.P., Ren W.M., Wang Y.M., Rao D.Y., Lu X.B. (2008). Asymmetric, region-and stereo-selective alternating copolymerization of CO_2_ and propylene oxide catalyzed by chiral chromium Salan complexes. J. Polym. Sci. Part A Polym. Chem..

[14] Wu G.P., Jiang S.D., Lu X.B., Ren W.M., Yan S.K. (2012). Stereoregular poly(cyclohexene carbonate)s: Unique crystallization behavior. Chin. J. Polym. Sci..

[15] Takahashi Y., Kojima R. (2003). Crystal structure of poly(trimethylene carbonate). Macromolecules.

[16] Shi L., Lu X.B., Zhang R., Peng X.J., Zhang C.Q., Li J.F., Peng X.M. (2006). Asymmetric alternating copolymerization and terpolymerization of epoxides with carbon dioxide at mild conditions. Macromolecules.

[17] Chen S.Y., Xiao M., Wang S.J., Han D.M., Meng Y.Z. (2012). Novel ternary block copolymerization of carbon dioxide with cyclohexene oxide and propylene oxide using zinc complex catalyst. J. Polym. Res..

[18] Seong J.E., Na S.J., Cyriac A., Kim B.W., Lee B.Y. (2010). Terpolymerizations of CO_2_, propylene oxide, and various epoxides using a Cobalt(III) complex of salen-type ligand tethered by four quaternary ammonium salts. Macromolecules.

[19] Song P.F., Xu H.D., Mao X.D., Liu X.J., Wang L. (2017). A one-step strategy for aliphatic poly(carbonate-ester)s with high performance derived from CO_2_, propylene oxide and l-lactide. Polym. Adv. Technol..

[20] Tao Y.H., Wang X.H., Zhao X.J., Li J., Wang F.S. (2006). Crosslinkable poly(propylene carbonate): High-yield synthesis and performance improvement. J. Polym. Sci. Part A Polym. Chem..

[21] Song P.F., Wang S.J., Xiao M., Du F.G., Gan L.Q., Liu G.Q., Meng Y.Z. (2009). Cross-linkable and thermally stable aliphatic polycarbonates derived from CO_2_, propylene oxide and maleic anhydride. J. Polym. Res..

[22] Cyriac A., Lee S.H., Lee B.Y. (2011). Connection of polymer chains using diepoxide in CO_2_/propylene oxide copolymerizations. Polym. Chem..

[23] Zeng S.S., Wang S.J., Xiao M., Han D.M., Meng Y.Z. (2011). Preparation and properties of biodegradable blend containing poly(propylene carbonate) and starch acetate with different degrees of substitution. Carbohydr. Polym..

[24] Kuang T.R., Li K.C., Chen B.Y., Peng X.F. (2017). Poly(propylene carbonate)-based in situ nanofibrillar biocomposites with enhanced miscibility, dynamic mechanical properties, rheological behavior and extrusion foaming ability. Compos. Part B Eng..

[25] Enriquez E., Mohanty A.K., Misra M. (2017). Biobased blends of poly(propylene carbonate) and poly(hydroxybutyrate-*co*-hydroxyvalerate): Fabrication and characterization. J. Appl. Polym. Sci..

[26] Chen S.S., Chen B., Fan J.S., Feng J.C. (2015). Exploring the application of sustainable poly(propylene carbonate) copolymer in toughening epoxy thermosets. ACS Sustain. Chem. Eng..

[27] Yang J., Pan H.W., Li X., Sun S.L., Zhang H.L., Dong L.S. (2017). A study on the mechanical, thermal properties and crystallization behavior of poly(lactic acid)/thermoplastic poly(propylene carbonate) polyurethane blends. RSC Adv..

[28] Gao J., Chen F., Wang K., Deng H., Zhang Q., Bai H.W., Fu Q. (2011). A promising alternative to conventional polyethylene with poly(propylene carbonate) reinforced by graphene oxide nanosheets. J. Mater. Chem..

[29] Bian J., Wei X.W., Lin H.L., Gong S.J., Zhang H., Guan Z.P. (2011). Preparation and characterization of modified graphite oxide/poly(propylene carbonate) composites by solution intercalation. Polym. Degrad. Stab..

[30] Shi X.D., Gan Z.H. (2007). Preparation and characterization of poly(propylene carbonate)/montmorillonite nanocomposites by solution intercalation. Eur. Polym. J..

[31] Zhai L.P., Li G.F., Xu Y., Xiao M., Wang S.J., Meng Y.Z. (2015). Poly(propylene carbonate)/aluminum flake composite films with enhanced gas barrier properties. J. Appl. Polym. Sci..

[32] Liao J.G., Li Y.Q., Zou Q., Duan X.Z., Yang Z.P., Xie Y.F., Liu H.H. (2016). Preparation, characterization and properties of nano-hydroxyapatite/polypropylene carbonate biocomposite. Mat. Sci. Eng. C.

[33] Chen L.J., Qin Y.S., Wang X.H., Zhao X.J., Wang F.S. (2011). Plasticizing while toughening and reinforcing poly(propylene carbonate) using low molecular weight urethane: Role of hydrogen-bonding interaction. Polymer.

[34] Qin Y.S., Chen L.J., Wang X.H., Zhao X.J., Wang F.S. (2011). Enhanced mechanical performance of poly(propylene carbonate) via hydrogen bonding interaction with o-lauroyl chitosan. Carbohydr. Polym..

[35] Wang X.L., Li R.K.Y., Cao Y.X., Meng Y.Z. (2007). Essential work of fracture analysis for starch filled poly(propylene carbonate) composites. Mater. Des..

[36] Qi X.D., Jing M.F., Liu Z.W., Dong P., Liu T.Y., Fu Q. (2016). Microfibrillated cellulose reinforced bio-based poly(propylene carbonate) with dual-responsive shape memory properties. RSC Adv..

[37] Wang Y.M., Song X.Y., Shao S.H., Xu P.X., Ren W.M., Lu X.B. (2013). Functionalization of carbon nanotubes by surface-initiated immortal alternating polymerization of CO_2_ and epoxides. Polym. Chem..

[38] Okada A., Kikuchi S., Yamada T. (2011). Alternating copolymerization of propylene oxide/alkylene oxide and carbon dioxide: Tuning thermal properties of polycarbonates. Chem. Lett..

[39] Tao Y.H., Wang X.H., Zhao X.J., Li J., Wang F.S. (2006). Double propagation based on diepoxide, a facile route to high molecular weight poly(propylene carbonate). Polymer.

[40] Han B., Zhang L., Zhang H.Y., Ding H.N., Liu B.Y., Wang X.H. (2016). One-pot synthesis and postpolymerization functionalization of cyclic carbonate/epoxide-difunctional polycarbonates prepared by regioselective diepoxide/CO_2_ copolymerization. Polym. Chem..

[41] Song P.F., Xiao M., Du F.G., Wang S.J., Gan L.Q., Liu G.Q., Meng Y.Z. (2008). Synthesis and properties of aliphatic polycarbonates derived from carbon dioxide, propylene oxide and maleic anhydride. J. Appl. Polym. Sci..

[42] Liu Y.F., Huang K.L., Peng D.M., Wu H. (2006). Synthesis, characterization and hydrolysis of an aliphatic polycarbonate by terpolymerization of carbon dioxide, propylene oxide and maleic anhydride. Polymer.

[43] Jeon J.Y., Eo S.C., Varghese J.K., Lee B.Y. (2014). Copolymerization and terpolymerization of carbon dioxide/propylene oxide/phthalic anhydride using a (salen)Co(III) complex tethering four quaternary ammonium salts. J. Org. Chem..

[44] Nörnberg B., Luinstra G.A. (2015). Influence of norbornene dicarboxylic anhydride on the copolymerization of carbon dioxide and propylene oxide. Eur. Polym. J..

[45] Duan Z.Y., Wang X.Y., Gao Q., Zhang L., Liu B.Y., Kim I. (2014). Highly active bifunctional cobalt-salen complexes for the synthesis of poly(ester-block-carbonate) copolymer via terpolymerization of carbon dioxide, propylene oxide, and norbornene anhydride isomer: Roles of anhydride conformation consideration. J. Polym. Sci. Part A Polym. Chem..

[46] Liu Y.L., Xiao M., Wang S.J., Xia L., Han D.M., Cui G.F., Meng Y.Z. (2014). Mechanism studies of terpolymerization of phthalic anhydride, propylene epoxide, and carbon dioxide catalyzed by ZnGA. RSC Adv..

[47] Gao L.J., Feng J.Y. (2013). A one-step strategy for thermally and mechanically reinforced pseudo-interpenetrating poly(propylene carbonate) networks by terpolymerization of CO_2_, propylene oxide and pyromellitic dianhydride. J. Mater. Chem. A..

[48] Song P.F., Mao X.D., Zhang X.F., Zhu X.G., Wang R.M. (2014). A one-step strategy for cross-linkable aliphatic polycarbonates with high degradability derived from CO_2_, propylene oxide and itaconic anhydride. RSC Adv..

[49] Hilf J., Scharfenberg M., Poon J., Moers C., Frey H. (2015). Aliphatic polycarbonates based on carbon dioxide, furfuryl glycidyl ether, and glycidyl methyl ether: Reversible functionalization and cross-linking. Macromol. Rapid. Commun..

[50] SDBSWeb. http://sdbs.db.aist.go.jp.

[51] Li X.H., Meng Y.Z., Zhu Q., Tjong S.C. (2003). Thermal decomposition characteristics of poly(propylene carbonate) using TG/IR and Py-GC/MS techniques. Polym. Degrad. Stab..

[52] Wu J.S., Xiao M., He H., Wang S.J., Han D.M., Meng Y.Z. (2011). Copolymerization of propylene oxide and carbon dioxide in the presence of diphenylmethane diisocyanate. J. Polym. Res..

