# Supplementary material for: Enhanced Poly(Propylene Carbonate) with Thermoplastic Networks: A One-Pot Synthesis from Carbon Dioxide, Propylene Oxide, and a Carboxylic Dianhydride
Source: Polymers (Basel). 2018 May 21;10(5):552. doi: 10.3390/polym10050552 (PMC6415432; doi:10.3390/polym10050552)
Supplement: Supplementary file 1 [file polymers-10-00552-s001.pdf]

Supporting Information for

Enhanced poly(propylene carbonate) with thermoplastic networks: A one-pot synthesis from carbon dioxide, propylene oxide and a carboxylic dianhydride

Xianggen Chen[1,2], Lingyun Wang[2], Jiuying Feng[1], Xianling Huang[1], Xiuzhi Guo[1], Jing Chen[1], Zhenyuan Xiao[1], Xiangjun Liang[1] and Lijun Gao[1,*]

[1] School of Chemistry and Chemical Engineering, Resource and Chemical Engineering Technology Research Center of Western Guangdong Province, Lingnan Normal University, Zhanjiang, P. R. China. 524048;

[2] Key Laboratory of Functional Molecular Engineering of Guangdong Province, School of Chemistry and Chemical Engineering, South China University of Technology, Guangzhou, P. R. China. 510641;

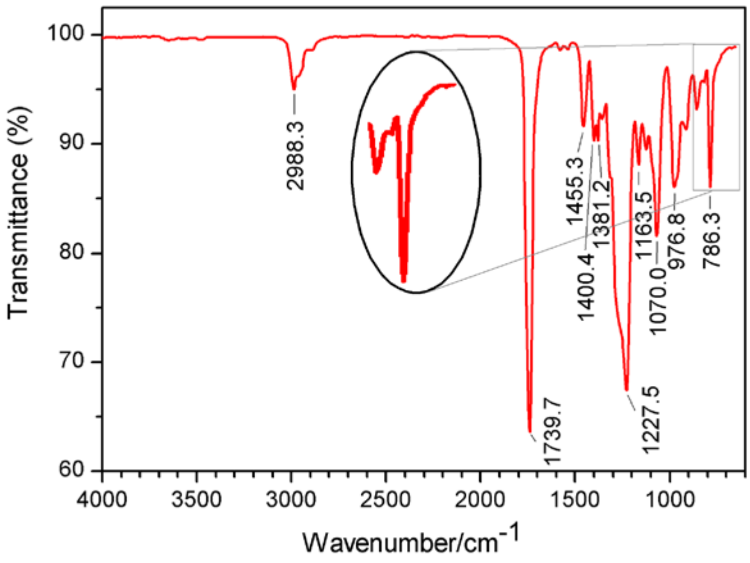

Figure S1. The FT-IR spectrum of PPC.

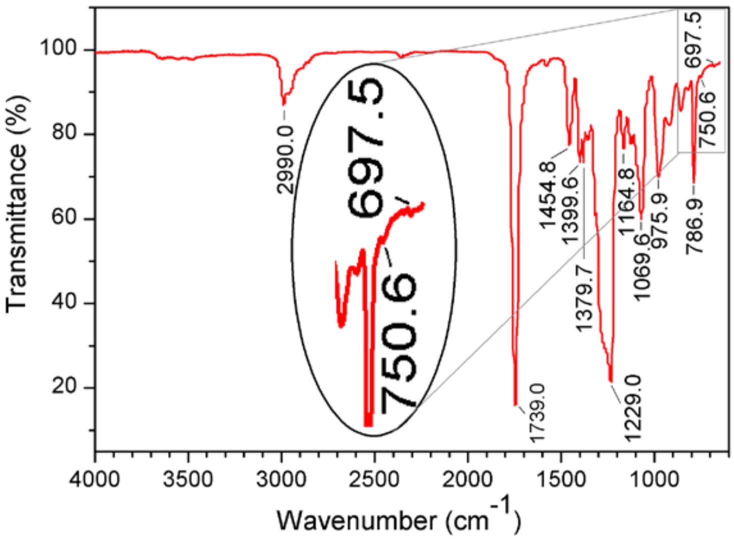

Figure S2. The FT-IR spectrum of PPC-3.

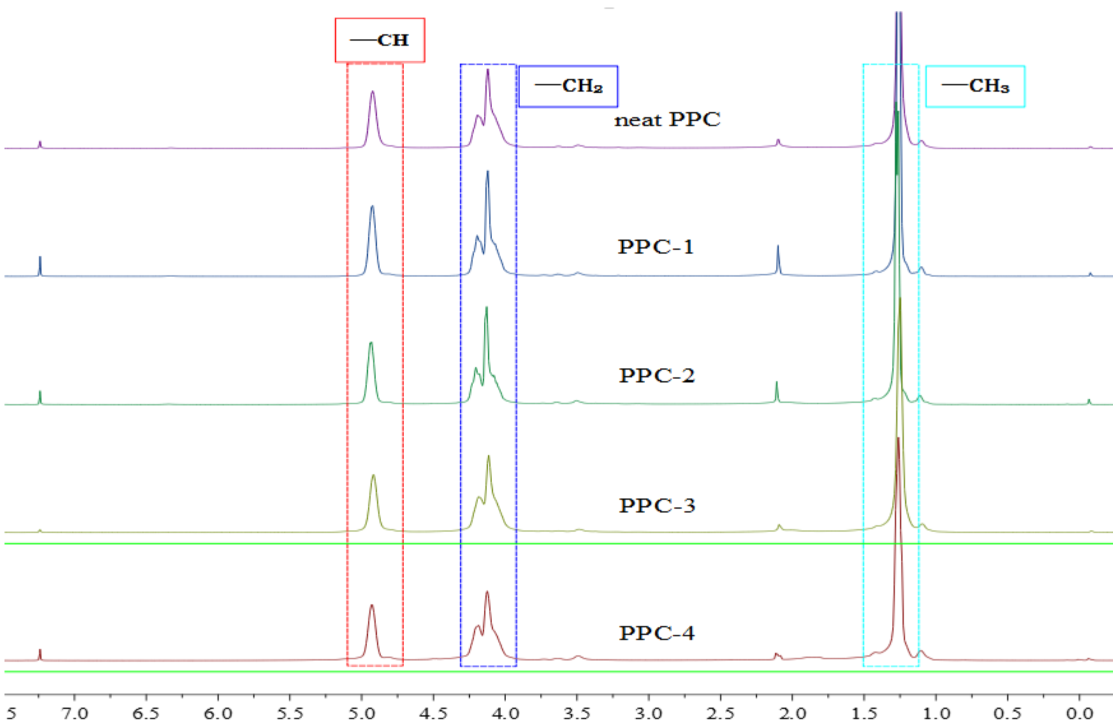

Figure S3. The ^{1}H NMR spectra of PPC and PPC with networks.

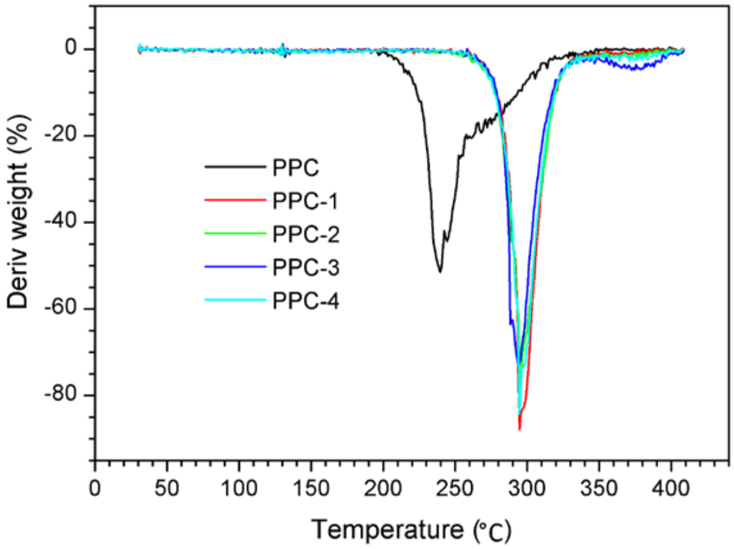

Figure S4. The DTG curves for PPC and PPC with networks.

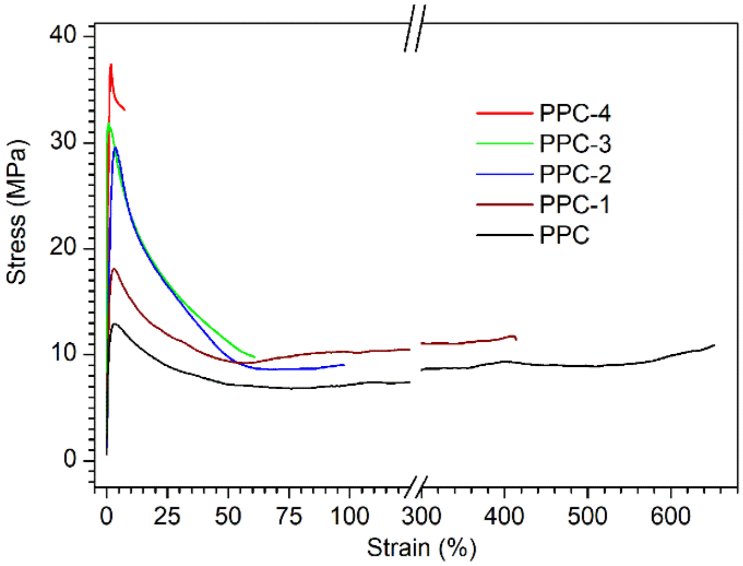

Figure S5. The strain-stress curves for PPC and PPC with networks.

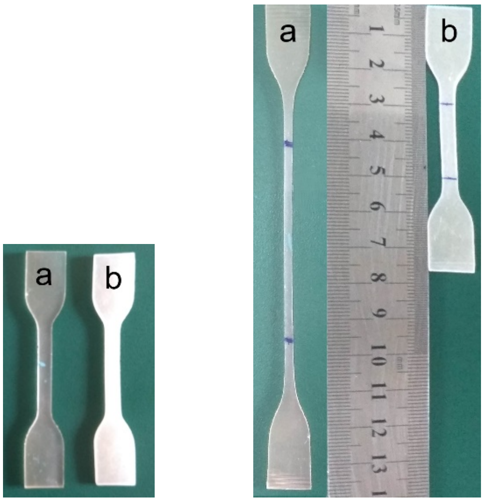

Figure S6. The photos of dumbbell-shaped specimens before (left) and after (right) hot-set test. (a) PPC, (b) PPC-4. The right photo is permanent deformation result.
